# Prehospital tranexamic acid in trauma patients: a systematic review and meta-analysis of randomized controlled trials

**DOI:** 10.3389/fmed.2023.1284016

**Published:** 2023-10-20

**Authors:** Pawan Acharya, Aamir Amin, Sandhya Nallamotu, Chaudhry Zaid Riaz, Venkataramana Kuruba, Virushnee Senthilkumar, Harika Kune, Sandeep Singh Bhatti, Iván Moguel Sarlat, Chekuri Vamsi Krishna, Kainat Asif, Abdulqadir J. Nashwan, Huzaifa Ahmad Cheema

**Affiliations:** ^1^Department of Trauma and Orthopedics, Lister Hospital, Stevenage, United Kingdom; ^2^Department of Cardiothoracic Surgery, Royal Brompton Hospital, London, United Kingdom; ^3^Kasturba Medical College, Manipal, Karnataka, India; ^4^Department of Medicine, Madinah Teaching Hospital, Faisalabad, Pakistan; ^5^Department of Orthopedics, All India Institute of Medical Sciences, Mangalagiri, Andhra Pradesh, India; ^6^Coimbatore Medical College, Coimbatore, Tamil Nadu, India; ^7^Kamineni Institute of Medical Sciences, Narketpally, Telangana, India; ^8^Manila Central University College of Medicine, Caloocan, Philippines; ^9^Servicio de Ortopedia y Traumatología, Hospital General Dr Agustín O’Horán, Mérida, Yucatán, Mexico; ^10^Alluri Sitaramraju Academy of Medical Sciences, Eluru, Andhra Pradesh, India; ^11^Department of Medicine, Dr. Ruth K. M. Pfau Civil Hospital Karachi, Karachi, Pakistan; ^12^Hamad Medical Corporation, Doha, Qatar; ^13^Department of Emergency Medicine, King Edward Medical University, Lahore, Pakistan

**Keywords:** prehospital TXA, out-of-hospital TXA, trauma, traumatic brain injury, meta-analysis

## Abstract

**Background:**

Prehospital tranexamic acid (TXA) may hold substantial benefits for trauma patients; however, the data underlying its efficacy and safety is scarce.

**Methods:**

We searched PubMed, Embase, the Cochrane Library, and ClinicalTrials.gov from inception to July 2023 for all randomized controlled trials (RCTs) investigating prehospital TXA in trauma patients as compared to placebo or standard care without TXA. Data were pooled under a random-effects model using RevMan 5.4 with risk ratio (RR) and mean difference (MD) as the effect measures.

**Results:**

A total of three RCTs were included in this review. Regarding the primary outcomes, prehospital TXA reduced the risk of 1-month mortality (RR 0.82, 95% CI 0.69–0.97) but did not increase survival with a favorable functional outcome at 6 months (RR 1.00, 95% CI 0.93–1.09). Prehospital TXA also reduced the risk of 24-h mortality but did not affect the risk of mortality due to bleeding and traumatic brain injury. There was no significant difference between the TXA and control groups in the incidence of RBC transfusion, and the number of ventilator- and ICU-free days. Prehospital TXA did not increase the risk of adverse events except for a small increase in the incidence of infections.

**Conclusion:**

Prehospital TXA is useful in reducing mortality in trauma patients without a notable increase in the risk of adverse events. However, there was no effect on the 6-month favorable functional status. Further large-scale trials are required to validate the aforementioned findings.

**Systematic review registration:**

PROSPERO (CRD42023451759).

## Introduction

In 2021, about 8% of global mortality (4.4 million) was injury-related (1). Contributing to death in 33–56% of traumatic fatalities in the pre-hospital setting, hemorrhage is a leading cause of preventable death, especially within the first 24 h of injury (2, 3). The significance of the prehospital period owing to its inherent vulnerability to derangements in the coagulation cascade has been highlighted in many clinical studies (4). Presenting within several minutes to a few hours after injury, the ensuing hyperfibrinolysis and hypocoagulation increase the risk of death due to bleeding (5, 6).

To combat trauma-induced coagulopathy and mitigate the effects of hemorrhage, several resuscitative adjuncts and hemostatic agents are currently in use worldwide (7). Among them, tranexamic acid (TXA) has gained popularity as a component of several protocols for major hemorrhage (8). First described in the 1960s, this anti-fibrinolytic is a lysine analog that binds plasminogen reversibly and inhibits fibrin dissolution, thus reducing hemorrhage (9, 10). The initial 2–3 h are imperative in deciding the burden of death caused by traumatic hemorrhage (11). Therefore, it is the early administration of TXA that confers the maximum benefits of anti-fibrinolytic therapy, which tends to decline over time (12).

The effect of in-hospital administration of TXA in trauma patients with significant hemorrhage was evaluated in the Clinical Randomization of an Antifibrinolytic in Significant Hemorrhage (CRASH)–2 and CRASH-3 trials (13, 14). Notwithstanding the significant reduction in mortality that is observed in suspected or clinically hemorrhaging trauma cases who received TXA in the aforementioned trials, the evidence on its prehospital use is fairly limited and scanty. A small number of randomized controlled trials (RCTs) have explored the safety and efficacy of prehospital TXA (15–17), but there have been very few systematic appraisals or meta-analyses of the evidence base (18, 19). Recently, the PATCH Trauma trial, the largest RCT to date to assess prehospital TXA in trauma patients, was published (20), therefore, necessitating an updated meta-analysis of all available data.

This systematic review and meta-analysis aims to increase the statistical power of the evidence by combining relevant studies to assess the effectiveness and safety of prehospital TXA for trauma patients.

## Methods

This study followed the standard Preferred Reporting Items for Systemic Review and Meta-analysis (PRISMA) guidelines (21). The study protocol is registered at PROSPERO (CRD42023451759).

### Data sources and search strategy

We searched MEDLINE (PubMed), Embase, the Cochrane Library, and ClinicalTrials.gov from inception to July 2023 using keywords and Medical Subject Headings (MeSH) relating to “trauma,” “tranexamic acid,” “traumatic brain injury,” “TXA,” “prehospital,” and “out-of-hospital.” A partial search of Google Scholar was also conducted to review any relevant grey literature. Additionally, we screened the reference lists of relevant studies to identify further eligible studies.

### Study selection and eligibility criteria

In order to find the relevant publications, two reviewers independently examined the search output and applied the eligibility criteria. Discussion with a third reviewer was used to settle any disagreements and conflicts between the two reviewers. The following inclusion and exclusion criteria were applied: (1) population: people who had suspected hemorrhage following traumatic injury; (2) intervention: administration of TXA in the prehospital settings; (3) comparison: placebo or standard care without TXA; and (4) study design: RCTs.

### Data extraction and outcomes

Data extraction was done using a pre-piloted Excel sheet. The data items of interest included authors, year, publication country, and characteristics of the study population and intervention (dose, route, and timing of TXA).

Our primary outcomes of interest were the risk of all-cause mortality at 1 month and survival with a favorable functional outcome at 6 months, as defined by a Glasgow Outcome Scale-Extended (GOS-E) score > 4. Our secondary outcomes consisted of the risk of 24-h all-cause mortality, mortality due to bleeding and traumatic brain injury (TBI), the number of patients receiving a red blood cell (RBC) transfusion, the number of ventilator- and ICU-free days, and the incidence of adverse events (AEs) and serious adverse events (SAEs). We also assessed specific AEs of interest including seizures, thromboembolic events, and infection or sepsis.

### Risk of bias assessment

The risk of bias in the included studies was assessed using the Cochrane Risk of Bias (RoB 1.0) tool (22). RoB 1.0 addresses seven specific domains: (1) random sequence generation; (2) allocation concealment; (3) blinding of the participants and personnel; (4) blinding of the outcome assessors; (5) incomplete outcome data; (6) selective outcome reporting; and (7) other source of bias. Each trial was rated as being at a low, unclear, or high risk of bias.

### Statistical analysis

The meta-analysis was performed using RevMan 5.4 software under a random-effects model due to the anticipated heterogeneity of the included RCTs. Our effect measures were risk ratios (RRs) and mean differences (MDs) with 95% confidence intervals (CIs) for dichotomous and continuous outcomes, respectively. The I_2_ statistic was used to quantify the heterogeneity of the analyses. We were unable to assess publication bias due to less than 10 studies being included in our review.

## Results

### Search results and characteristics of included studies

**Figure 1:**
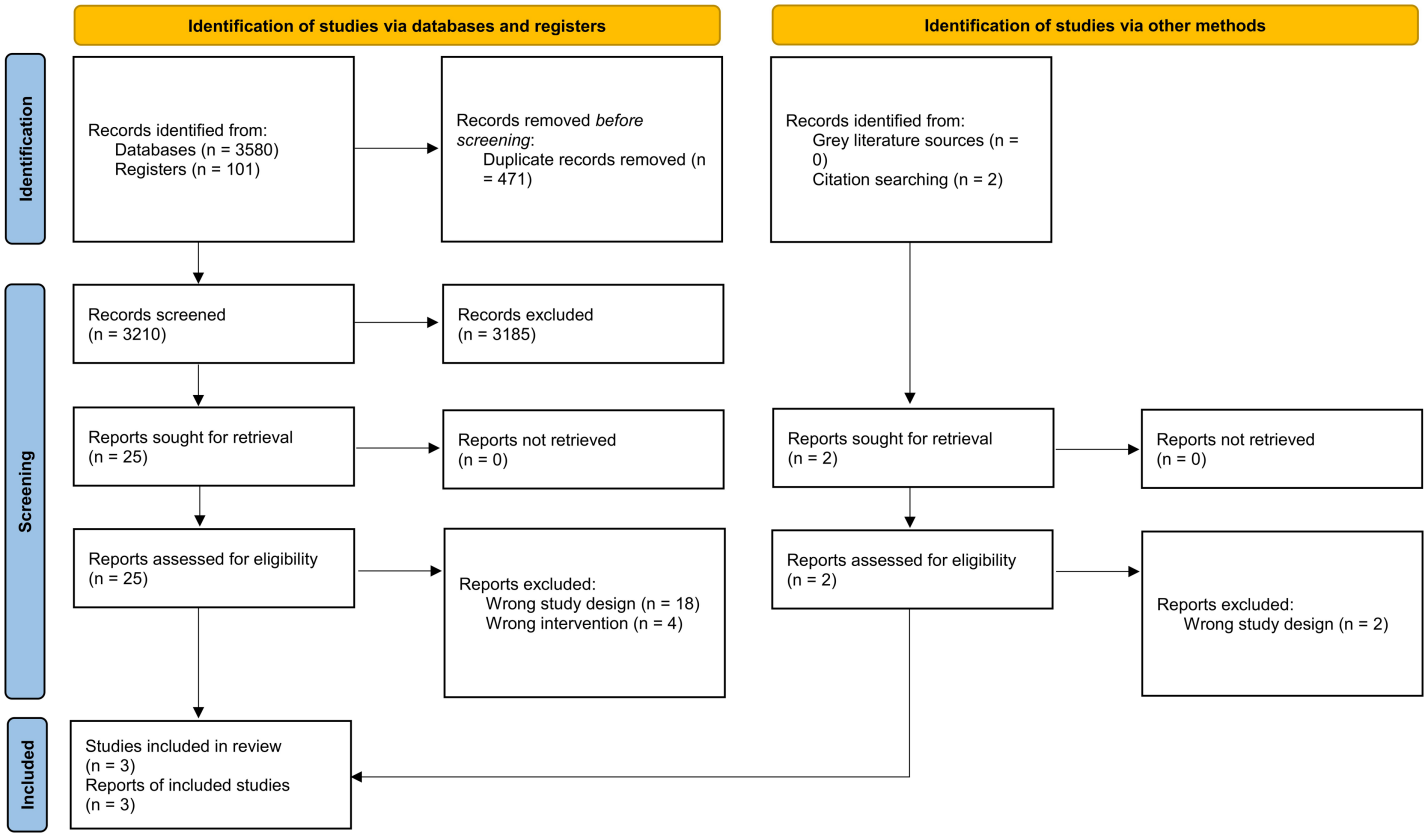
PRISMA 2020 flowchart of the study selection process.

After screening, a total of three RCTs were included in our review (15, 17, 20). The detailed screening process is depicted through a PRISMA flowchart (Figure 1).

Three studies were conducted in the year 2020 (15, 17) and one study was conducted in 2023 (20). The studies were conducted in the USA, Australia, New Zealand, and Canada. The dose and regimen of prehospital TXA varied between the studies. One study enrolled TBI patients exclusively (17). The characteristics of included RCTs are summarized in Supplementary Table S1.

### Risk of bias in included studies

**Figure 2:**
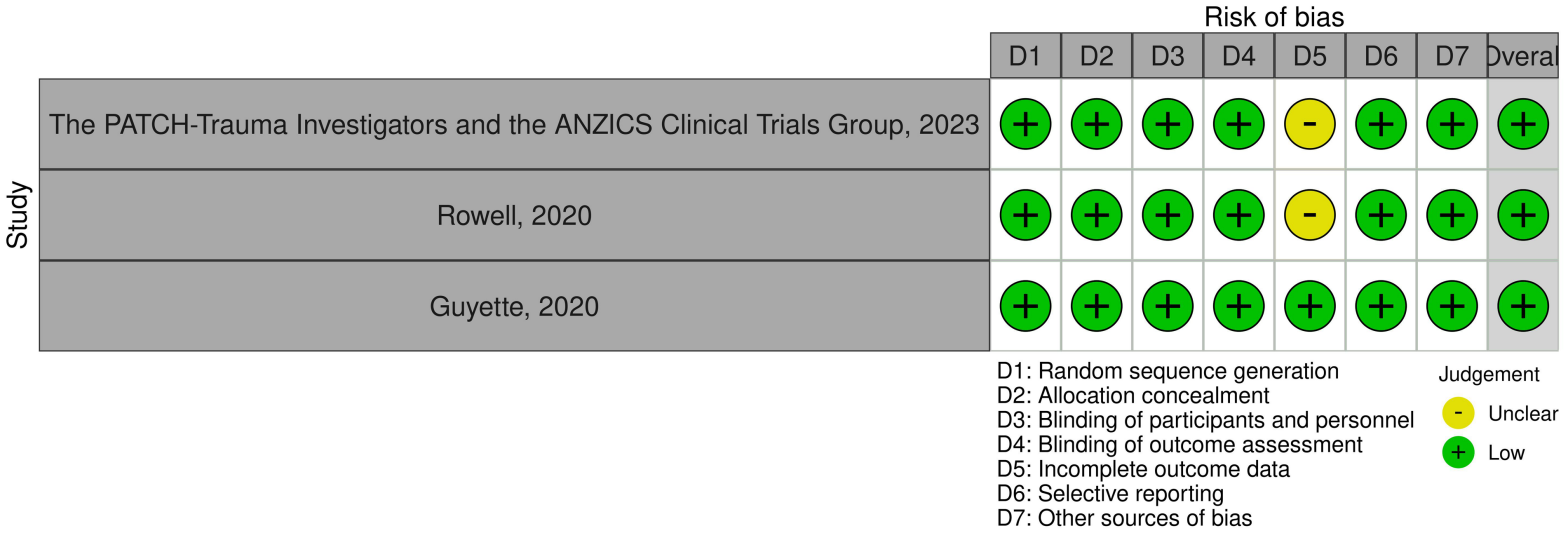
Quality assessment of included trials.

Overall, all three studies were found to have a low risk of bias. There was an unclear risk of bias in the domain of incomplete outcome data in two studies (17, 20); apart from that, all other domains had a low risk of bias. The complete risk of bias assessment is illustrated in Figure 2.

## Results of meta-analysis

### Primary outcomes

#### 1-month mortality

**Figure 3:**
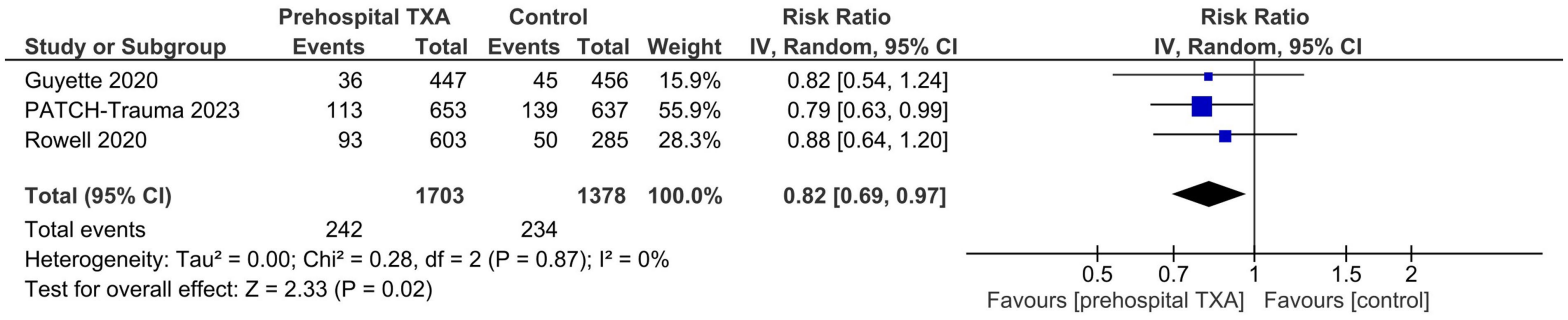
Effect of prehospital tranexamic acid on 1-month all-cause mortality in trauma patients.

All three trials reported all-cause mortality at 1 month. Our meta-analysis showed that prehospital TXA reduced the risk of 1-month mortality as compared to the control group (RR 0.82, 95% CI 0.69–0.97; Figure 3). The estimated heterogeneity was low (I^2^ = 0%).

#### Survival with a favorable functional outcome at 6 months (GOS-E > 4)

**Figure 4:**
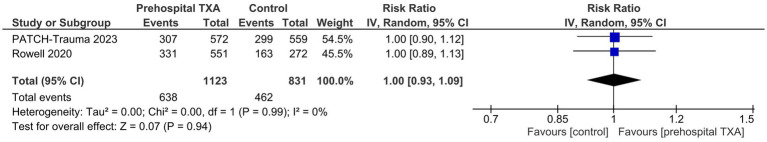
Effect of prehospital tranexamic acid on survival with a favorable functional outcome at 6 months in trauma patients.

Pooled results from two studies showed that there was no significant difference in survival with a favorable functional outcome at 6 months between the prehospital TXA and control groups (RR 1.00, 95% CI 0.93–1.09, I^2^ = 0%; Figure 4).

### Secondary outcomes

Our meta-analysis indicated that prehospital TXA reduced the risk of 24-h mortality (RR 0.73, 95% CI 0.56–0.96, I^2^ = 0%; Supplementary Figure S1). There was no significant difference in the risk of mortality due to bleeding (RR 0.74, 95% CI 0.43–1.29, I^2^ = 12%; Supplementary Figure S2) and TBI (RR 0.87, 95% CI 0.69–1.10, I^2^ = 0; Supplementary Figure S3), and the incidence of RBC transfusion (RR 0.88, 95% CI 0.69–1.14, I^2^ = 69%; Supplementary Figure S4). In addition, prehospital TXA did not increase the number of ventilator-free days (MD 1.06 days, 95% CI −0.79 to 2.92, I^2^ = 61%; Supplementary Figure S5).

Prehospital TXA was not associated with an increase in the number of overall AEs (RR 0.90, 95% CI 0.45–1.81, I^2^ = 74%; Supplementary Figure S6) and SAEs (RR 0.68, 95% CI 0.44–1.06, I^2^ = 0%; Supplementary Figure S7). There was no significant difference between the prehospital TXA and control groups in the incidence of seizures (RR 1.14, 95% CI 0.59–2.20, I^2^ = 0%; Supplementary Figure S8) and thromboembolic events (RR 1.04, 95% CI 0.72–1.49, I^2^ = 62%; Supplementary Figure S9). However, prehospital TXA increased the risk of infection or sepsis (RR 1.17, 95% CI 1.03–1.33, I^2^ = 0%; Supplementary Figure S10).

## Discussion

To the best of our knowledge, this is the largest meta-analysis to date that examines the effect of prehospital TXA in trauma patients. Our analysis showed that prehospital TXA reduced the risk of all-cause 1-month mortality but had no effect on functional recovery at 6 months. Additionally, prehospital TXA reduced the risk of mortality at 24 h. However, there was no difference between the two groups in terms of mortality due to bleeding or TBI, the number of patients that received an RBC infusion, and the number of ventilator- and ICU-free days. Prehospital TXA was not associated with an increase in overall adverse events, seizures, and thromboembolic events but it did slightly increase the risk of infection or sepsis.

The results of our meta-analysis are in accordance with the findings of a prior meta-analysis which showed a reduction in 24-h and 1-month mortality rates (18). Likewise, TXA administration in trauma patients did not increase the risk of thromboembolic events or seizures compared to the control group. However, the validity of the comparison of our results with this meta-analysis is questionable because although the authors aimed to investigate prehospital administration of TXA (18), the most contribution to their analyses was from the CRASH-2 and CRASH-3 trials (13, 14) in which TXA was initiated in the in-hospital rather than the prehospital settings. Two other previous systematic reviews also attempted to address the concerned research question (19, 23); however, one included only one RCT (19) while the other did not quantitatively pool the relevant studies (23). Therefore, our meta-analysis is the first to focus on evidence from randomized controlled studies to evaluate the role of TXA started in the prehospital setting in an unbiased manner.

Our findings are consistent with those reported from the in-hospital settings. The CRASH-2 trial was the first landmark trial that demonstrated a reduction in mortality rates in trauma patients with TXA administration (13). This trial also showed a decreased risk of death from bleeding in line with the mechanism of TXA (13). It is unclear why the same was not observed in our meta-analysis; however, this is likely due to a lack of adequate power as only two trials reported this outcome. Future trials should ameliorate this concern, and conclusively establish the role of prehospital TXA in trauma patients. Additionally, the mortality benefit while highly encouraging should be interpreted in the context of no increase in survival with favorable functional outcome at 6 months. This is consistent with the PATCH-Trauma and Prehospital TXA for TBI trials which also found no increase in favorable functional status (17, 20). Nevertheless, the lack of improvement in functional outcome may be due to a lack of power, and hence, requires corroboration from future RCTs.

The rationale behind the use of prehospital TXA is that the greatest benefit of TXA is seen with early administration of TXA, especially ≤1 h after injury (24, 25). Conversely, late administration of TXA, especially after 3 h of injury, may even increase the risk of mortality owing to a higher likelihood of adverse events, particularly disseminated intravascular coagulation and uncontrolled hemorrhage (24, 25). Most of the patients pooled from the RCTs included in our meta-analysis received TXA within 2 h after injury. However, we were unable to conduct a subgroup analysis to investigate the benefit of earlier administration of TXA due to the lack of availability of necessary data from the included RCTs. An individual patient-level meta-analysis may be able to better address this question in the future.

A concerning find in this meta-analysis is there was a higher risk of infections with TXA, albeit the increase was of a small magnitude. This may need to be further examined in future studies to support or refute our observation. More importantly, our analysis reassuringly demonstrated no increase in seizures or thromboembolic events, adverse events that have been of particular concern in trials of TXA for various indications (16, 20, 26–29).

### Strength and limitations

The main strength of our meta-analysis is that all our studies were found to have a low risk of bias. Additionally, we only focused on RCTs and excluded observational studies to prevent our results from being limited by confounding bias.

There are, however, a few limitations to our meta-analysis. Our study was an aggregate-level meta-analysis, incorporating only three RCTs due to a focused research question. Therefore, we were unable to investigate potential effect modifiers such as the timing of TXA administration, and type of trauma population. Additionally, we witnessed differences in the dosage and regimen of TXA across the trials which is a potential source of heterogeneity. Future RCTs should also attempt to investigate whether pre- and in-hospital administration of TXA has any incremental benefits over in-hospital administration alone, especially in countries with advanced trauma systems.

## Conclusion

The results of our meta-analysis evaluating prehospital TXA use in trauma patients indicate that prehospital TXA is useful in reducing mortality in trauma patients without a notable increase in the risk of adverse events. However, there was no effect on the 6-month favorable functional status. Further large-scale, well-designed, randomized, multicenter trials are required to validate the aforementioned findings and conclusively determine the effectiveness of prehospital administration of TXA.

## Data availability statement

The original contributions presented in the study are included in the article/Supplementary material, further inquiries can be directed to the corresponding authors.

## Author contributions

PA: Conceptualization, Investigation, Validation, Visualization, Writing – review & editing. AA: Conceptualization, Investigation, Validation, Visualization, Writing – original draft. SN: Conceptualization, Data curation, Formal analysis, Validation, Writing – original draft. CR: Formal analysis, Software, Writing – original draft. VS: Data curation, Formal analysis, Writing – original draft. HK: Data curation, Formal analysis, Writing – original draft. SB: Data curation, Formal analysis, Writing – original draft. IS: Data curation, Investigation, Writing – review & editing. CK: Investigation, Writing – review & editing. KA: Visualization, Writing – review & editing. AN: Conceptualization, Funding acquisition, Project administration, Writing – review & editing. HC: Conceptualization, Methodology, Project administration, Software, Supervision, Validation, Writing – review & editing. VK: _.
