# Supplementary material for: Prehospital tranexamic acid in trauma patients: a systematic review and meta-analysis of randomized controlled trials
Source: Front Med (Lausanne). 2023 Oct 20;10:1284016. doi: 10.3389/fmed.2023.1284016 (PMC10623347; doi:10.3389/fmed.2023.1284016)

# Supplementary Material

**Prehospital tranexamic acid in trauma patients: a systematic review and meta-analysis of randomized controlled trials**

This supplemental material has been provided by the authors to give readers additional information about their work.

**Table S1.** Characteristics of included studies.

| **Study ID** | **Trial name** | **Location** | **Sample size** | **Population** | **Age (years)** | **Male (%)** | **Type of injury (%)** | **ISS** | **Score >2 on the AIS for head or neck** | **GCS score** | **Intervention and comparator** |
| --- | --- | --- | --- | --- | --- | --- | --- | --- | --- | --- | --- |
| The PATCH-Trauma Investigators and the ANZICS Clinical Trials Group, 2023 | PATCH-Trauma | Australia, New Zealand | 1300 (657 vs. 643) | Adults (≥18 years of age) with suspected severe traumatic injuries who were treated at the scene by paramedics or physicians and transported by road or air ambulance to participating trauma centers, and were at high risk for trauma-induced coagulopathy. | 44.1±19.7 vs. 44.2±18.9 | 69.9 vs. 71.4 | Blunt: 92.8 vs. 91.4 Penetrating: 6.7 vs. 8.6 Burn: 0.5 vs. 0 | 29.0 (18.0–41.0) vs. 29.0 (17.0–38.0) | 40.9 vs. 37.7 | 13.0 (6.0 to 14.0) 13.0 (6.0 to 14.0) | One dose of TXA (1g) or placebo intravenously as a bolus (utilizing a slow-push method over 10 minutes) as soon as practicable at the scene or en route to the receiving hospital. After hospital arrival, the second 10-ml ampule containing 1g TXA or placebo was added to 1 liter of 0.9% sodium chloride solution and infused over a period of 8 hours. |
| Rowell, 2020^1^ | Prehospital TXA for TBI | USA, Canada | 966 (312 vs. 345 vs. 309) | Patients aged 15 years or older with moderate or severe blunt or penetrating TBI, a GCS score of 3 to 12, at least 1 reactive pupil, and systolic blood pressure of at least 90 mm Hg before randomization. | 39 (26-57) vs. 40 (26-56) vs. 36 (25-55) | 73 vs. 74 vs. 76 | Blunt: 97 vs. 98 vs. 95 Penetrating: 4 vs. 1 vs. 5 | 17 (8-27) vs. 17 (8-27) vs. 17 (9-27) | 56 vs. 56 vs. 59 | 7.8 (3.3) vs. 7.8 (3.3) vs. 7.6 (3.2) | 1g IV TXA bolus in the out-of-hospital setting followed by a 1g TXA IV infusion initiated upon hospital arrival and infused over 8 hours (bolus maintenance group), 2-g IV TXA bolus in the out-of-hospital setting followed by a placebo infusion (bolus only group), or IV placebo bolus in the out-of-hospital setting followed by an IV placebo infusion (placebo group). |
| Guyette, 2020 | STAAMP | USA | 927 (460 vs. 467) | Injured patients at risk for hemorrhage transported from the scene or transferred from an outside emergency department to a participating site within an estimated 2 hours of the time of injury were eligible for enrollment if they experienced at least 1 episode of hypotension (systolic blood pressure ≤90 mmHg) or tachycardia (heart rate ≥110 beats per minute) before arrival at a participating center. | 41±17 vs. 42±18 | 73.2 vs. 74.8 | Blunt: 83.0 vs. 85.3 Penetrating: 17.4 vs. 15.4 | 13 (5-22) vs. 11 (4-22 | 26.0 vs. 23.5 | - | TXA:   Phase A (pre-hospital: 1 g TXA in 10 mL solution or 10 mL sterile water placebo and infused for 10 min)   Phase B (in-hospital: 1 g TXA in 10 mL solution or 10 mL placebo (sterile water) and infused for 10 min)   Phase C (in-hospital: 1 g TXA in 10 mL solution or 10 mL placebo and infused for 8 h)   The abbreviated dosing regimen was 1 g TXA bolus (phase A), placebo bolus (phase B), and placebo infusion (phase C). The standard dosing regimen was 1 g TXA bolus (phase A), placebo bolus (phase B), and 1 g TXA infusion (phase C). The repeat bolus dosing regimen was 1 g TXA bolus (phase A), 1 g TXA bolus (phase B), and 1 g TXA infusion (phase C)   Placebo: placebo bolus (phase A), placebo bolus (phase B), and placebo infusion (phase C) |
| ISS, Injury Severity Score; AIS, Abbreviated Injury Scale; TXA, tranexamic acid; GCS, Glasgow Coma Scale; RBC, red blood cell.  ^1^Data are for bolus maintenance TXA group vs. bolus only TXA group vs. placebo. | | | | | | | | | | | |

**Figure S1.** Effect of prehospital tranexamic acid on 24-hour mortality in trauma patients.


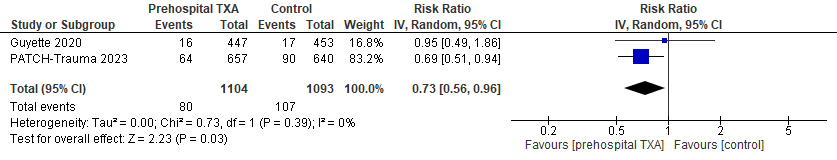


**Figure S2.** Effect of prehospital tranexamic acid on mortality due to bleeding in trauma patients.


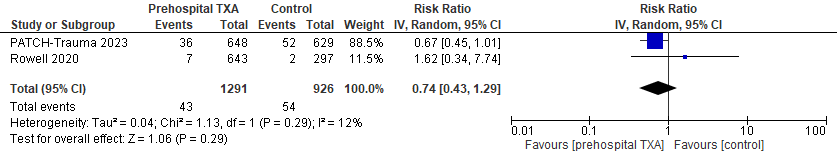


**Figure S3.** Effect of prehospital tranexamic acid on mortality due to traumatic brain injury in trauma patients.


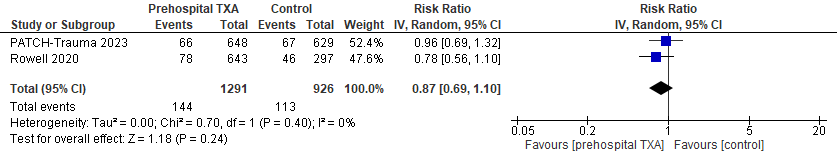


**Figure S4.** Effect of prehospital tranexamic acid on the incidence of RBC transfusion in trauma patients.


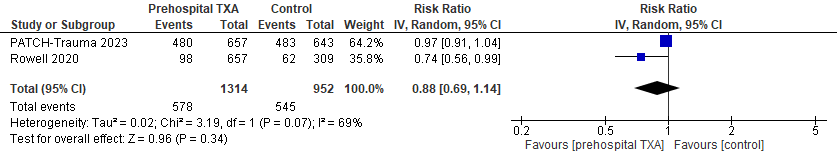


**Figure S5.** Effect of prehospital tranexamic acid on the number of ventilator-free days in trauma patients.


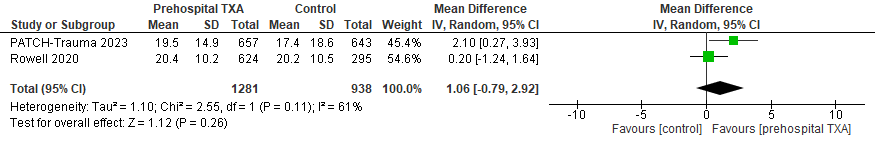


**Figure S6.** Effect of prehospital tranexamic acid on overall adverse events in trauma patients.


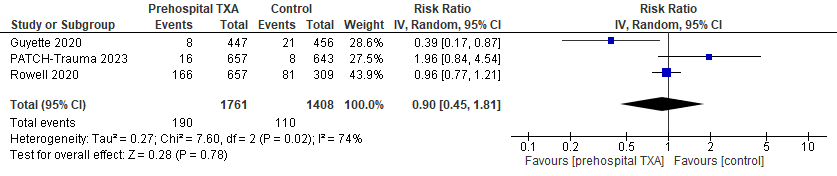


**Figure S7.** Effect of prehospital tranexamic acid on serious adverse events in trauma patients.


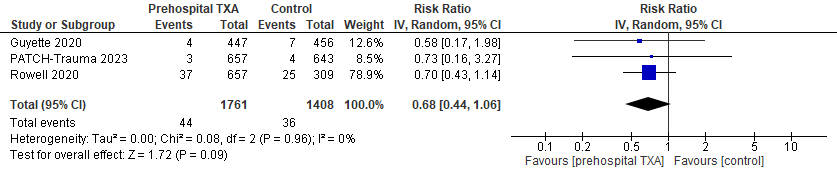


**Figure S8.** Effect of prehospital tranexamic acid on the incidence of seizures in trauma patients.


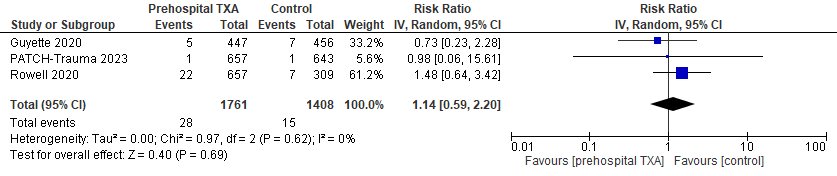


**Figure S9.** Effect of prehospital tranexamic acid on the incidence of thromboembolic events in trauma patients.


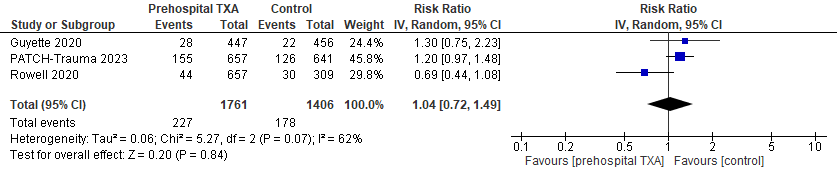


**Figure S10.** Effect of prehospital tranexamic acid on the risk of infection or sepsis in trauma patients.


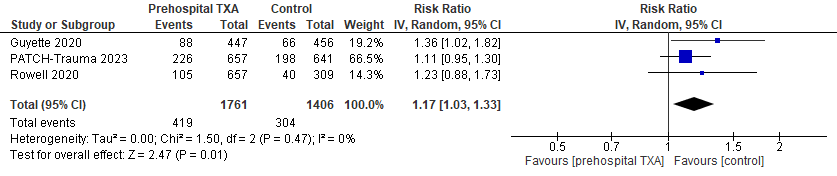

Supplement: Supplementary file 1 [file Data_Sheet_1.docx]
